# *rac*-5′′-(4-Fluoro­benzyl­idene)-1′-(4-fluoro­phen­yl)-1′′-methyl-1′,2′,3′,5′,6′,7′,8′,8a′-octa­hydro­dispiro­[acenaphthyl­ene-1,3′-indolizine-2′,3′′-piperidine]-2,4′′(1*H*)-dione

**DOI:** 10.1107/S1600536812051094

**Published:** 2012-12-22

**Authors:** J. Suresh, R. A. Nagalakshmi, S. Sivakumar, R. Ranjith Kumar, P. L. Nilantha Lakshman

**Affiliations:** a Department of Physics, The Madura College, Madurai 625 011, India; b Department of Organic Chemistry, School of Chemistry, Madurai Kamaraj University, Madurai 625 021, India; c Department of Food Science and Technology, University of Ruhuna, Mapalana, Kamburupitiya 81100, Sri Lanka

## Abstract

In the title *E* isomer of the racemic compound, C_37_H_32_F_2_N_2_O_2_, the pyridinone ring adopts a twisted half-chair conformation with the N atom deviating by −0.355 (3) Å and with the methyl­ene C atom next to octa­hydro­indolizine moiety deviating by 0.415 (3) Å from the mean plane defined by other four atoms. In the octa­hydro­indolizine system, the pyrrolidine ring exhibits an envelope conformation with the fused methyne C atom deviating by 0.6496 (1) Å from the mean plane defined by four other atoms, and the piperidine ring exhibits a distorted chair conformation as evident from the puckering parameters *Q* = 0.568 (2) Å, θ = 1.0 (2) and Φ = 256 (11)°. In the crystal, C—H⋯O inter­actions connect the mol­ecules into chains along [101].

## Related literature

For general properties of indolizines, see: Malonne *et al.* (1998 ▶); Medda *et al.* (2003 ▶); Pearson & Guo (2001 ▶). For related structures, see: Sussman & Wodak (1973 ▶); Wodak (1975 ▶). For ring conformation analysis, see: Cremer & Pople (1975 ▶).
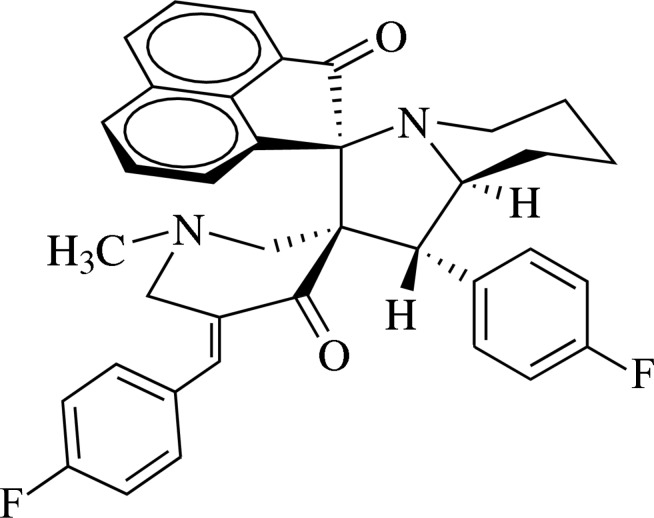


## Experimental

### 

#### Crystal data

C_37_H_32_F_2_N_2_O_2_
*M* *_r_* = 574.65
Monoclinic, 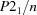

*a* = 10.2716 (4) Å
*b* = 20.0353 (7) Å
*c* = 14.3790 (6) Å
β = 97.047 (1)°
*V* = 2936.8 (2) Å^3^
*Z* = 4
Mo *K*α radiation
μ = 0.09 mm^−1^
*T* = 293 K
0.21 × 0.19 × 0.18 mm

#### Data collection

Bruker Kappa APEXII diffractometer
Absorption correction: multi-scan (*SADABS*; Sheldrick, 1996 ▶) *T*_min_ = 0.967, *T*_max_ = 0.974
33177 measured reflections
7519 independent reflections
4823 reflections with *I* > 2σ(*I*)
*R*_int_ = 0.033

#### Refinement

*R*[*F* ^2^ > 2σ(*F* ^2^)] = 0.046
*wR*(*F* ^2^) = 0.126
*S* = 1.01
7519 reflections
388 parameters
H-atom parameters constrained
Δρ_max_ = 0.22 e Å^−3^
Δρ_min_ = −0.21 e Å^−3^

### 

Data collection: *APEX2* (Bruker, 2004 ▶); cell refinement: *SAINT* (Bruker, 2004 ▶); data reduction: *SAINT*; program(s) used to solve structure: *SHELXS97* (Sheldrick, 2008 ▶); program(s) used to refine structure: *SHELXL97* (Sheldrick, 2008 ▶); molecular graphics: *PLATON* (Spek, 2009 ▶); software used to prepare material for publication: *SHELXL97*.

## Supplementary Material

Crystal structure: contains datablock(s) global, I. DOI: 10.1107/S1600536812051094/ld2088sup1.cif

Structure factors: contains datablock(s) I. DOI: 10.1107/S1600536812051094/ld2088Isup2.hkl

Additional supplementary materials:  crystallographic information; 3D view; checkCIF report

## Figures and Tables

**Table 1:** Hydrogen-bond geometry (Å, °)

*D*—H⋯*A*	*D*—H	H⋯*A*	*D*⋯*A*	*D*—H⋯*A*
C11—H11*A*⋯O1^i^	0.97	2.49	3.352 (2)	148

Symmetry code: (i) 
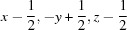
.

**Fig. 1.**
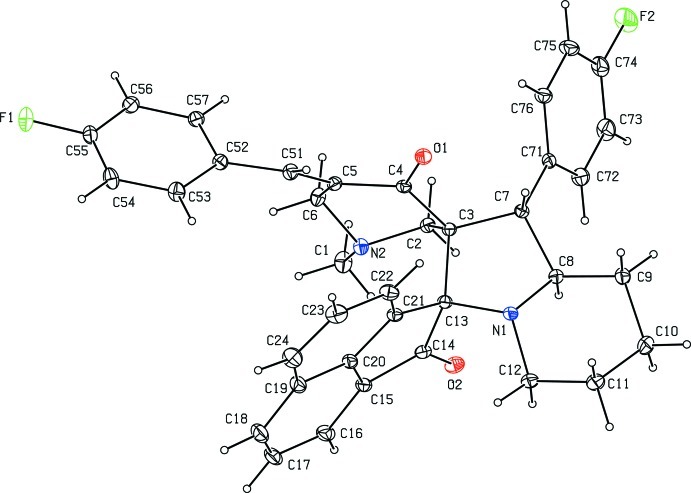
The molecular structure of (I), showing 20% probability displacement ellipsoids and the atom-numbering scheme.

**Fig. 2.**
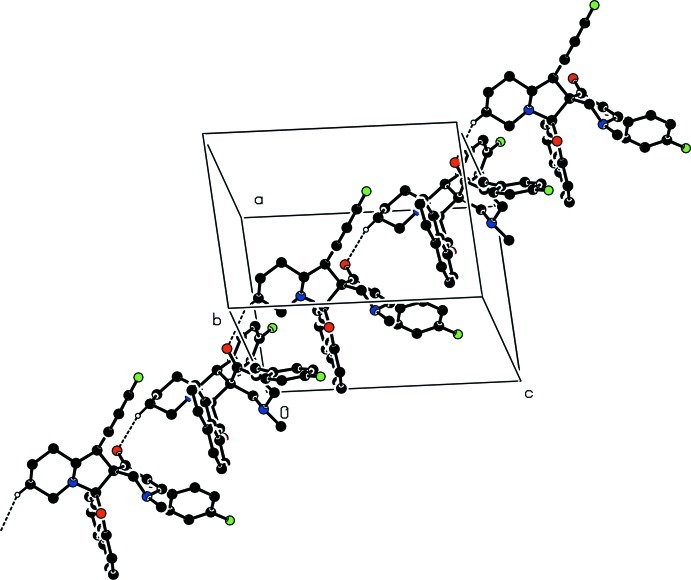
Partial packing diagram showing C—H···O interactions.

## supplementary crystallographic information

### Comment

Indolizine derivatives have been found to possess a variety of biological activities such as anti-inflammatory (Malonne *et al.*, 1998), antiviral (Medda *et al.*, 2003) and anti-tumor (Pearson & Guo, 2001) activities. In view of its medicinal importance and in conjunction with our research interests, we synthesized the title compound and report here its X-ray structure.

In the title compound (Fig.1), the pyridinone ring adopts twisted half chair conformation with atoms N2 and C2 deviating by -0.355 (3)Å and 0.415 (3)Å respectively, from the mean plane defined by other atoms C3/C4/C5/C6. The sum of bond angles around N2 (332.65 (1) °) indicates a pyramidal geometry. Although the atoms C1, C2, C6 attached to the atom N2, are all in Sp^2^ hybridization, their different environments cause differences in bond lengths (N2-C2 (1.4467 (19) Å) and N2-C6 (1.457 (2) Å)) and in the bond angles (C1-N2-C2 (112.35 (13) °), C1-N2-C6 (111.00 (14) °) and C2-N2-C6 (109.31 (12) °)). The methyl group at position 1 of the pyridinone ring is in equatorial orientation, denoted by the torsion angle C1-N2-C6-C5 (177.38 (1) °). In the fused system , the pyrrolidine ring adopts the twisted envelope conformation with C8 atom at the flap deviating by 0.6496 (1) Å from the mean plane defined by other atoms C7/C3/C13/N1 and this orientation may be due to the intra-molecular C7—H7···O1 interaction. In the fused system the piperidine ring adopts a slightly distorted chair conformation as evident from the puckering parameters Q = 0.568 (2) Å, θ = 1.0 (2)° and Φ = 256 (11)° (Cremer & Pople, 1975). The twist of the 4-fluorobenzene ring (C52-C57) with respect to the spiro junction is denoted by the torsion angle C5-C51-C52-C57 (-49.1 (2) °). The dihedral angle between the mean plane of the pyridinone ring, defined by the atoms C2/C4/C5/C6 with the two 4-fluorobenzene rings are 87.70 (1) ° and 63.20 (1)°. The carbonyl bond length, i.e C4=O1 (1.214 (2) Å), is somewhat longer, due to C—H···O contacts. The C8—N1 bond length (1.456 (2) Å) is comparable with the C*Sp*^2^—N*Sp*^2^ distance found in similar structures (Sussman & Wodak, 1973; Wodak, 1975).

The structure is stabilized by intermolecular C11—H11A···O1 interactions generating chains along [101] (Fig. 2).

### Experimental

A mixture of 1-methyl-3,5-bis[(*E*)-4-fluromethylidene]tetrahydro-4(1*H*)- pyridinone (1 mmol), acenaphthenequinone (1 mmol) and piperidine-2- carboxylic acid (1 mmol) was dissolved in isopropyl alcohol (15 ml) and heated to reflux for 60 min. After completion of the reaction, as evident from TLC, the mixture was poured into water (50 ml), the precipitated solid was filtered and washed with water (100 ml) to obtain pure yellow solid. Melting point:498 K, Yield: 93%

### Refinement

H atoms were placed at calculated positions and allowed to ride on their carrier atoms with C—H = 0.93–0.98 Å; *U*_iso_ = 1.2*U*_eq_(C) for CH_2_ and CH groups, and *U*_iso_ = 1.5*U*_eq_(C) for CH_3_ groups. The (0 1 1) reflection was probably affected by the beam-stop and was omitted from the refinement.

### Crystal data

**Table:**

C_37_H_32_F_2_N_2_O_2_	*F*(000) = 1208
*M**_r_* = 574.65	*D*_x_ = 1.300 Mg m^−^^3^
Monoclinic, *P*2_1_/*n*	Mo *K*α radiation, λ = 0.71073 Å
Hall symbol: -P 2yn	Cell parameters from 2000 reflections
*a* = 10.2716 (4) Å	θ = 2–31°
*b* = 20.0353 (7) Å	µ = 0.09 mm^−^^1^
*c* = 14.3790 (6) Å	*T* = 293 K
β = 97.047 (1)°	Block, yellow
*V* = 2936.8 (2) Å^3^	0.21 × 0.19 × 0.18 mm
*Z* = 4	

### Data collection

**Table:**

Bruker Kappa APEXII diffractometer	7519 independent reflections
Radiation source: fine-focus sealed tube	4823 reflections with *I* > 2σ(*I*)
Graphite monochromator	*R*_int_ = 0.033
Detector resolution: 0 pixels mm^-1^	θ_max_ = 28.6°, θ_min_ = 2.0°
ω and φ scans	*h* = −13→13
Absorption correction: multi-scan (*SADABS*; Sheldrick, 1996)	*k* = −27→27
*T*_min_ = 0.967, *T*_max_ = 0.974	*l* = −19→19
33177 measured reflections	

### Refinement

**Table:**

Refinement on *F*^2^	Primary atom site location: structure-invariant direct methods
Least-squares matrix: full	Secondary atom site location: difference Fourier map
*R*[*F*^2^ > 2σ(*F*^2^)] = 0.046	Hydrogen site location: inferred from neighbouring sites
*wR*(*F*^2^) = 0.126	H-atom parameters constrained
*S* = 1.01	*w* = 1/[σ^2^(*F*_o_^2^) + (0.0467*P*)^2^ + 0.8383*P*] where *P* = (*F*_o_^2^ + 2*F*_c_^2^)/3
7519 reflections	(Δ/σ)_max_ < 0.001
388 parameters	Δρ_max_ = 0.22 e Å^−^^3^
0 restraints	Δρ_min_ = −0.21 e Å^−^^3^

### Special details

**Table:**

Geometry. All e.s.d.'s (except the e.s.d. in the dihedral angle between two l.s. planes) are estimated using the full covariance matrix. The cell e.s.d.'s are taken into account individually in the estimation of e.s.d.'s in distances, angles and torsion angles; correlations between e.s.d.'s in cell parameters are only used when they are defined by crystal symmetry. An approximate (isotropic) treatment of cell e.s.d.'s is used for estimating e.s.d.'s involving l.s. planes.
Refinement. Refinement of F^2^ against ALL reflections. The weighted R -factor wR and goodness of fit S are based on F^2^, conventional R-factors R are based on F, with F set to zero for negative F^2^. The threshold expression of (F^2^ > 2σ(F^2^) is used only for calculating R-factors(gt) etc. and is not relevant to the choice of reflections for refinement. R -factors based on F^2^ are statistically about twice as large as those based on F, and R- factors based on ALL data will be even larger.

### Fractional atomic coordinates and isotropic or equivalent isotropic displacement parameters (Å^2^)

**Table:**

	*x*	*y*	*z*	*U*_iso_*/*U*_eq_	
O1	0.61115 (10)	0.19654 (5)	0.39944 (8)	0.0470 (3)	
N1	0.39601 (12)	0.29516 (7)	0.21792 (9)	0.0406 (3)	
N2	0.30062 (12)	0.28413 (6)	0.49902 (9)	0.0409 (3)	
C3	0.45465 (13)	0.28569 (7)	0.38350 (10)	0.0333 (3)	
C2	0.39870 (15)	0.32425 (7)	0.46112 (11)	0.0378 (3)	
H2A	0.4686	0.3352	0.5104	0.045*	
H2B	0.3596	0.3656	0.4362	0.045*	
O2	0.18118 (12)	0.35533 (6)	0.31669 (9)	0.0557 (3)	
C5	0.44596 (14)	0.18822 (7)	0.49900 (11)	0.0375 (3)	
C4	0.51377 (14)	0.22096 (7)	0.42563 (10)	0.0355 (3)	
F1	0.30715 (12)	−0.05223 (6)	0.77515 (9)	0.0769 (4)	
C13	0.34404 (13)	0.26706 (7)	0.29939 (10)	0.0358 (3)	
C7	0.55514 (14)	0.32673 (7)	0.33483 (11)	0.0363 (3)	
H7	0.6170	0.2946	0.3136	0.044*	
C14	0.20618 (15)	0.29644 (8)	0.31472 (11)	0.0410 (4)	
C71	0.63601 (15)	0.37673 (7)	0.39573 (11)	0.0390 (3)	
C20	0.17940 (15)	0.18241 (8)	0.29535 (11)	0.0434 (4)	
C51	0.45644 (15)	0.12233 (8)	0.50714 (12)	0.0433 (4)	
H51	0.5001	0.1009	0.4627	0.052*	
C52	0.40751 (15)	0.07919 (7)	0.57780 (12)	0.0414 (4)	
C21	0.31177 (14)	0.19344 (8)	0.28438 (11)	0.0391 (3)	
C8	0.47433 (15)	0.35371 (8)	0.24735 (11)	0.0420 (4)	
H8	0.4176	0.3901	0.2638	0.050*	
C6	0.36509 (17)	0.23063 (8)	0.55591 (12)	0.0479 (4)	
H6A	0.2995	0.2032	0.5805	0.057*	
H6B	0.4210	0.2496	0.6085	0.057*	
C56	0.39704 (17)	0.05049 (9)	0.73925 (13)	0.0514 (4)	
H56	0.4141	0.0606	0.8027	0.062*	
C57	0.43161 (16)	0.09393 (8)	0.67207 (12)	0.0478 (4)	
H57	0.4721	0.1341	0.6907	0.057*	
C15	0.11258 (15)	0.24044 (9)	0.31307 (11)	0.0449 (4)	
C22	0.38379 (17)	0.14106 (9)	0.25929 (13)	0.0509 (4)	
H22	0.4703	0.1472	0.2482	0.061*	
C72	0.60036 (19)	0.44278 (9)	0.40225 (14)	0.0574 (5)	
H72	0.5223	0.4576	0.3690	0.069*	
C1	0.21719 (19)	0.32362 (9)	0.55275 (15)	0.0599 (5)	
H1A	0.1537	0.2951	0.5764	0.090*	
H1B	0.1727	0.3572	0.5131	0.090*	
H1C	0.2701	0.3447	0.6042	0.090*	
C54	0.30853 (19)	−0.02399 (9)	0.61792 (15)	0.0588 (5)	
H54	0.2654	−0.0636	0.6002	0.071*	
C76	0.75231 (16)	0.35654 (9)	0.44646 (12)	0.0490 (4)	
H76	0.7783	0.3122	0.4436	0.059*	
C16	−0.01960 (17)	0.23783 (11)	0.32096 (13)	0.0597 (5)	
H16	−0.0663	0.2761	0.3323	0.072*	
C53	0.34488 (18)	0.01952 (8)	0.55166 (13)	0.0538 (4)	
H53	0.3272	0.0088	0.4885	0.065*	
C55	0.33711 (16)	−0.00765 (8)	0.70981 (14)	0.0497 (4)	
F2	0.86998 (16)	0.50943 (7)	0.55739 (10)	0.1067 (5)	
C12	0.30442 (17)	0.30500 (10)	0.13328 (12)	0.0555 (5)	
H12A	0.2417	0.3395	0.1438	0.067*	
H12B	0.2565	0.2640	0.1173	0.067*	
C19	0.11871 (18)	0.11956 (10)	0.28474 (13)	0.0575 (5)	
C9	0.55224 (19)	0.37571 (11)	0.16992 (13)	0.0612 (5)	
H9A	0.6165	0.3418	0.1601	0.073*	
H9B	0.5988	0.4167	0.1882	0.073*	
C75	0.83110 (19)	0.40116 (12)	0.50153 (14)	0.0656 (5)	
H75	0.9091	0.3871	0.5356	0.079*	
C23	0.3254 (2)	0.07716 (9)	0.25032 (15)	0.0655 (5)	
H23	0.3760	0.0412	0.2352	0.079*	
C17	−0.08141 (19)	0.17526 (13)	0.31121 (15)	0.0726 (7)	
H17	−0.1706	0.1726	0.3166	0.087*	
C73	0.6783 (2)	0.48739 (10)	0.45719 (16)	0.0703 (6)	
H73	0.6530	0.5317	0.4614	0.084*	
C18	−0.0162 (2)	0.11856 (13)	0.29418 (15)	0.0728 (6)	
H18	−0.0613	0.0783	0.2886	0.087*	
C74	0.7921 (2)	0.46553 (11)	0.50471 (14)	0.0673 (6)	
C11	0.3801 (2)	0.32515 (12)	0.05371 (13)	0.0710 (6)	
H11A	0.3192	0.3339	−0.0020	0.085*	
H11B	0.4370	0.2888	0.0399	0.085*	
C10	0.4618 (2)	0.38711 (13)	0.07917 (14)	0.0785 (7)	
H10A	0.4044	0.4247	0.0867	0.094*	
H10B	0.5137	0.3976	0.0291	0.094*	
C24	0.1984 (2)	0.06633 (10)	0.26295 (15)	0.0704 (6)	
H24	0.1640	0.0234	0.2571	0.085*	

### Atomic displacement parameters (Å^2^)

**Table:**

	*U*^11^	*U*^22^	*U*^33^	*U*^12^	*U*^13^	*U*^23^
O1	0.0377 (6)	0.0464 (6)	0.0572 (7)	0.0083 (5)	0.0073 (5)	0.0054 (5)
N1	0.0363 (6)	0.0506 (8)	0.0334 (7)	−0.0070 (6)	−0.0015 (5)	0.0022 (6)
N2	0.0427 (7)	0.0342 (7)	0.0471 (8)	0.0041 (5)	0.0112 (6)	−0.0001 (6)
C3	0.0329 (7)	0.0312 (7)	0.0348 (8)	0.0000 (6)	0.0001 (6)	0.0000 (6)
C2	0.0423 (8)	0.0318 (7)	0.0385 (8)	0.0003 (6)	0.0015 (7)	−0.0012 (6)
O2	0.0534 (7)	0.0513 (7)	0.0606 (8)	0.0169 (6)	−0.0003 (6)	0.0027 (6)
C5	0.0378 (7)	0.0356 (8)	0.0382 (8)	0.0005 (6)	0.0006 (6)	0.0017 (6)
C4	0.0334 (7)	0.0343 (8)	0.0368 (8)	−0.0007 (6)	−0.0030 (6)	−0.0036 (6)
F1	0.0915 (8)	0.0641 (7)	0.0801 (8)	−0.0139 (6)	0.0300 (7)	0.0206 (6)
C13	0.0309 (7)	0.0386 (8)	0.0369 (8)	0.0005 (6)	0.0002 (6)	0.0003 (6)
C7	0.0338 (7)	0.0359 (8)	0.0382 (8)	−0.0021 (6)	0.0009 (6)	0.0002 (6)
C14	0.0370 (8)	0.0490 (9)	0.0355 (8)	0.0059 (7)	−0.0008 (6)	0.0013 (7)
C71	0.0398 (8)	0.0383 (8)	0.0388 (8)	−0.0073 (6)	0.0040 (7)	0.0013 (6)
C20	0.0413 (8)	0.0525 (10)	0.0352 (8)	−0.0091 (7)	−0.0003 (7)	−0.0011 (7)
C51	0.0459 (8)	0.0370 (8)	0.0471 (9)	0.0026 (7)	0.0060 (7)	0.0010 (7)
C52	0.0417 (8)	0.0314 (8)	0.0511 (10)	0.0020 (6)	0.0054 (7)	0.0009 (7)
C21	0.0374 (7)	0.0420 (8)	0.0362 (8)	−0.0025 (6)	−0.0020 (6)	−0.0035 (6)
C8	0.0411 (8)	0.0435 (9)	0.0397 (9)	−0.0050 (7)	−0.0011 (7)	0.0045 (7)
C6	0.0583 (10)	0.0403 (9)	0.0472 (10)	0.0054 (7)	0.0150 (8)	0.0049 (7)
C56	0.0553 (10)	0.0495 (10)	0.0482 (10)	−0.0011 (8)	0.0018 (8)	0.0038 (8)
C57	0.0501 (9)	0.0380 (8)	0.0528 (10)	−0.0064 (7)	−0.0043 (8)	0.0026 (7)
C15	0.0342 (8)	0.0641 (11)	0.0356 (8)	−0.0031 (7)	0.0016 (6)	0.0011 (7)
C22	0.0479 (9)	0.0498 (10)	0.0531 (10)	0.0015 (8)	−0.0012 (8)	−0.0127 (8)
C72	0.0640 (11)	0.0411 (10)	0.0646 (12)	−0.0049 (8)	−0.0023 (9)	0.0009 (8)
C1	0.0653 (11)	0.0504 (11)	0.0696 (13)	0.0120 (9)	0.0300 (10)	0.0006 (9)
C54	0.0687 (12)	0.0400 (9)	0.0706 (13)	−0.0163 (8)	0.0208 (10)	−0.0075 (9)
C76	0.0442 (9)	0.0558 (10)	0.0453 (10)	−0.0037 (7)	−0.0009 (7)	−0.0030 (8)
C16	0.0393 (9)	0.0945 (15)	0.0459 (10)	−0.0019 (9)	0.0078 (8)	0.0022 (10)
C53	0.0679 (11)	0.0416 (9)	0.0529 (11)	−0.0070 (8)	0.0113 (9)	−0.0083 (8)
C55	0.0465 (9)	0.0421 (9)	0.0631 (12)	0.0001 (7)	0.0164 (8)	0.0098 (8)
F2	0.1306 (12)	0.1010 (11)	0.0827 (10)	−0.0629 (9)	−0.0102 (9)	−0.0266 (8)
C12	0.0488 (9)	0.0757 (13)	0.0387 (9)	−0.0100 (9)	−0.0075 (8)	0.0055 (9)
C19	0.0574 (11)	0.0648 (12)	0.0486 (10)	−0.0234 (9)	−0.0004 (9)	−0.0006 (9)
C9	0.0575 (11)	0.0785 (13)	0.0465 (10)	−0.0232 (10)	0.0018 (8)	0.0115 (9)
C75	0.0523 (11)	0.0899 (16)	0.0509 (11)	−0.0152 (10)	−0.0089 (9)	−0.0055 (10)
C23	0.0784 (14)	0.0469 (11)	0.0673 (13)	0.0027 (10)	−0.0068 (11)	−0.0180 (9)
C17	0.0425 (10)	0.119 (2)	0.0568 (13)	−0.0283 (12)	0.0080 (9)	0.0046 (12)
C73	0.0943 (16)	0.0439 (11)	0.0718 (14)	−0.0180 (10)	0.0065 (12)	−0.0088 (10)
C18	0.0616 (12)	0.0925 (17)	0.0635 (13)	−0.0370 (12)	0.0041 (10)	−0.0013 (12)
C74	0.0825 (14)	0.0662 (13)	0.0521 (12)	−0.0346 (11)	0.0039 (11)	−0.0132 (10)
C11	0.0660 (12)	0.1065 (18)	0.0376 (10)	−0.0204 (12)	−0.0053 (9)	0.0102 (10)
C10	0.0806 (14)	0.1072 (18)	0.0451 (11)	−0.0291 (13)	−0.0032 (10)	0.0243 (11)
C24	0.0858 (15)	0.0498 (11)	0.0715 (14)	−0.0212 (11)	−0.0065 (12)	−0.0115 (10)

### Geometric parameters (Å, º)

**Table:**

O1—C4	1.2142 (17)	C57—H57	0.9300
N1—C8	1.456 (2)	C15—C16	1.377 (2)
N1—C12	1.457 (2)	C22—C23	1.413 (3)
N1—C13	1.4588 (19)	C22—H22	0.9300
N2—C2	1.4467 (19)	C72—C73	1.382 (3)
N2—C1	1.456 (2)	C72—H72	0.9300
N2—C6	1.457 (2)	C1—H1A	0.9600
C3—C4	1.5255 (19)	C1—H1B	0.9600
C3—C2	1.526 (2)	C1—H1C	0.9600
C3—C7	1.552 (2)	C54—C55	1.358 (3)
C3—C13	1.5987 (19)	C54—C53	1.376 (3)
C2—H2A	0.9700	C54—H54	0.9300
C2—H2B	0.9700	C76—C75	1.387 (2)
O2—C14	1.2084 (19)	C76—H76	0.9300
C5—C51	1.329 (2)	C16—C17	1.404 (3)
C5—C4	1.486 (2)	C16—H16	0.9300
C5—C6	1.499 (2)	C53—H53	0.9300
F1—C55	1.3587 (19)	F2—C74	1.356 (2)
C13—C21	1.521 (2)	C12—C11	1.515 (3)
C13—C14	1.574 (2)	C12—H12A	0.9700
C7—C71	1.511 (2)	C12—H12B	0.9700
C7—C8	1.519 (2)	C19—C24	1.403 (3)
C7—H7	0.9800	C19—C18	1.409 (3)
C14—C15	1.476 (2)	C9—C10	1.523 (3)
C71—C72	1.379 (2)	C9—H9A	0.9700
C71—C76	1.382 (2)	C9—H9B	0.9700
C20—C15	1.390 (2)	C75—C74	1.353 (3)
C20—C19	1.405 (2)	C75—H75	0.9300
C20—C21	1.405 (2)	C23—C24	1.356 (3)
C51—C52	1.469 (2)	C23—H23	0.9300
C51—H51	0.9300	C17—C18	1.356 (3)
C52—C57	1.380 (2)	C17—H17	0.9300
C52—C53	1.387 (2)	C73—C74	1.352 (3)
C21—C22	1.358 (2)	C73—H73	0.9300
C8—C9	1.514 (2)	C18—H18	0.9300
C8—H8	0.9800	C11—C10	1.518 (3)
C6—H6A	0.9700	C11—H11A	0.9700
C6—H6B	0.9700	C11—H11B	0.9700
C56—C55	1.361 (2)	C10—H10A	0.9700
C56—C57	1.379 (2)	C10—H10B	0.9700
C56—H56	0.9300	C24—H24	0.9300
			
C8—N1—C12	114.26 (13)	C21—C22—C23	119.06 (17)
C8—N1—C13	108.73 (12)	C21—C22—H22	120.5
C12—N1—C13	117.42 (12)	C23—C22—H22	120.5
C2—N2—C1	112.35 (13)	C71—C72—C73	121.29 (18)
C2—N2—C6	109.31 (12)	C71—C72—H72	119.4
C1—N2—C6	111.00 (14)	C73—C72—H72	119.4
C4—C3—C2	107.90 (12)	N2—C1—H1A	109.5
C4—C3—C7	112.03 (11)	N2—C1—H1B	109.5
C2—C3—C7	113.10 (12)	H1A—C1—H1B	109.5
C4—C3—C13	108.25 (11)	N2—C1—H1C	109.5
C2—C3—C13	112.02 (11)	H1A—C1—H1C	109.5
C7—C3—C13	103.47 (11)	H1B—C1—H1C	109.5
N2—C2—C3	109.64 (12)	C55—C54—C53	118.44 (16)
N2—C2—H2A	109.7	C55—C54—H54	120.8
C3—C2—H2A	109.7	C53—C54—H54	120.8
N2—C2—H2B	109.7	C71—C76—C75	121.25 (18)
C3—C2—H2B	109.7	C71—C76—H76	119.4
H2A—C2—H2B	108.2	C75—C76—H76	119.4
C51—C5—C4	117.46 (14)	C15—C16—C17	117.6 (2)
C51—C5—C6	124.01 (15)	C15—C16—H16	121.2
C4—C5—C6	118.47 (13)	C17—C16—H16	121.2
O1—C4—C5	121.20 (13)	C54—C53—C52	120.95 (18)
O1—C4—C3	121.49 (14)	C54—C53—H53	119.5
C5—C4—C3	117.30 (12)	C52—C53—H53	119.5
N1—C13—C21	110.80 (12)	C54—C55—F1	118.30 (16)
N1—C13—C14	113.10 (12)	C54—C55—C56	123.00 (17)
C21—C13—C14	101.45 (12)	F1—C55—C56	118.68 (17)
N1—C13—C3	102.78 (11)	N1—C12—C11	109.17 (14)
C21—C13—C3	117.13 (12)	N1—C12—H12A	109.8
C14—C13—C3	112.01 (12)	C11—C12—H12A	109.8
C71—C7—C8	116.73 (12)	N1—C12—H12B	109.8
C71—C7—C3	116.03 (12)	C11—C12—H12B	109.8
C8—C7—C3	103.64 (11)	H12A—C12—H12B	108.3
C71—C7—H7	106.6	C24—C19—C20	116.23 (17)
C8—C7—H7	106.6	C24—C19—C18	128.21 (19)
C3—C7—H7	106.6	C20—C19—C18	115.51 (19)
O2—C14—C15	127.07 (15)	C8—C9—C10	110.59 (15)
O2—C14—C13	124.46 (14)	C8—C9—H9A	109.5
C15—C14—C13	107.99 (13)	C10—C9—H9A	109.5
C72—C71—C76	117.69 (15)	C8—C9—H9B	109.5
C72—C71—C7	122.95 (14)	C10—C9—H9B	109.5
C76—C71—C7	119.35 (14)	H9A—C9—H9B	108.1
C15—C20—C19	123.18 (16)	C74—C75—C76	118.64 (19)
C15—C20—C21	113.29 (14)	C74—C75—H75	120.7
C19—C20—C21	123.46 (16)	C76—C75—H75	120.7
C5—C51—C52	127.93 (15)	C24—C23—C22	122.47 (19)
C5—C51—H51	116.0	C24—C23—H23	118.8
C52—C51—H51	116.0	C22—C23—H23	118.8
C57—C52—C53	118.16 (16)	C18—C17—C16	122.57 (18)
C57—C52—C51	121.22 (14)	C18—C17—H17	118.7
C53—C52—C51	120.41 (16)	C16—C17—H17	118.7
C22—C21—C20	118.31 (15)	C74—C73—C72	118.93 (19)
C22—C21—C13	132.12 (14)	C74—C73—H73	120.5
C20—C21—C13	109.48 (13)	C72—C73—H73	120.5
N1—C8—C9	110.11 (14)	C17—C18—C19	121.27 (19)
N1—C8—C7	100.39 (12)	C17—C18—H18	119.4
C9—C8—C7	115.35 (13)	C19—C18—H18	119.4
N1—C8—H8	110.2	C73—C74—C75	122.20 (18)
C9—C8—H8	110.2	C73—C74—F2	119.1 (2)
C7—C8—H8	110.2	C75—C74—F2	118.7 (2)
N2—C6—C5	110.76 (13)	C12—C11—C10	110.83 (18)
N2—C6—H6A	109.5	C12—C11—H11A	109.5
C5—C6—H6A	109.5	C10—C11—H11A	109.5
N2—C6—H6B	109.5	C12—C11—H11B	109.5
C5—C6—H6B	109.5	C10—C11—H11B	109.5
H6A—C6—H6B	108.1	H11A—C11—H11B	108.1
C55—C56—C57	117.91 (17)	C11—C10—C9	110.19 (17)
C55—C56—H56	121.0	C11—C10—H10A	109.6
C57—C56—H56	121.0	C9—C10—H10A	109.6
C56—C57—C52	121.51 (16)	C11—C10—H10B	109.6
C56—C57—H57	119.2	C9—C10—H10B	109.6
C52—C57—H57	119.2	H10A—C10—H10B	108.1
C16—C15—C20	119.84 (17)	C23—C24—C19	120.37 (18)
C16—C15—C14	132.40 (17)	C23—C24—H24	119.8
C20—C15—C14	107.67 (13)	C19—C24—H24	119.8
			
C1—N2—C2—C3	−163.25 (14)	C12—N1—C8—C7	179.61 (13)
C6—N2—C2—C3	73.06 (15)	C13—N1—C8—C7	46.28 (15)
C4—C3—C2—N2	−58.85 (14)	C71—C7—C8—N1	−170.67 (13)
C7—C3—C2—N2	176.66 (11)	C3—C7—C8—N1	−41.77 (14)
C13—C3—C2—N2	60.20 (15)	C71—C7—C8—C9	71.06 (19)
C51—C5—C4—O1	−27.6 (2)	C3—C7—C8—C9	−160.05 (14)
C6—C5—C4—O1	155.33 (15)	C2—N2—C6—C5	−58.13 (17)
C51—C5—C4—C3	151.39 (14)	C1—N2—C6—C5	177.38 (14)
C6—C5—C4—C3	−25.72 (19)	C51—C5—C6—N2	−141.71 (16)
C2—C3—C4—O1	−145.48 (14)	C4—C5—C6—N2	35.2 (2)
C7—C3—C4—O1	−20.35 (19)	C55—C56—C57—C52	−0.5 (3)
C13—C3—C4—O1	93.10 (15)	C53—C52—C57—C56	1.1 (3)
C2—C3—C4—C5	35.57 (16)	C51—C52—C57—C56	−173.68 (15)
C7—C3—C4—C5	160.70 (12)	C19—C20—C15—C16	0.0 (3)
C13—C3—C4—C5	−85.85 (15)	C21—C20—C15—C16	176.90 (15)
C8—N1—C13—C21	−156.45 (12)	C19—C20—C15—C14	−177.03 (15)
C12—N1—C13—C21	71.89 (17)	C21—C20—C15—C14	−0.10 (19)
C8—N1—C13—C14	90.42 (14)	O2—C14—C15—C16	−6.2 (3)
C12—N1—C13—C14	−41.24 (19)	C13—C14—C15—C16	−178.49 (17)
C8—N1—C13—C3	−30.55 (14)	O2—C14—C15—C20	170.25 (16)
C12—N1—C13—C3	−162.21 (14)	C13—C14—C15—C20	−2.01 (17)
C4—C3—C13—N1	−116.32 (13)	C20—C21—C22—C23	−3.5 (3)
C2—C3—C13—N1	124.84 (12)	C13—C21—C22—C23	−179.66 (17)
C7—C3—C13—N1	2.70 (14)	C76—C71—C72—C73	0.3 (3)
C4—C3—C13—C21	5.37 (17)	C7—C71—C72—C73	−178.59 (17)
C2—C3—C13—C21	−113.47 (14)	C72—C71—C76—C75	−0.5 (3)
C7—C3—C13—C21	124.39 (13)	C7—C71—C76—C75	178.47 (16)
C4—C3—C13—C14	121.97 (13)	C20—C15—C16—C17	0.5 (3)
C2—C3—C13—C14	3.13 (17)	C14—C15—C16—C17	176.62 (17)
C7—C3—C13—C14	−119.01 (13)	C55—C54—C53—C52	−1.0 (3)
C4—C3—C7—C71	−90.36 (15)	C57—C52—C53—C54	−0.4 (3)
C2—C3—C7—C71	31.85 (17)	C51—C52—C53—C54	174.44 (16)
C13—C3—C7—C71	153.26 (12)	C53—C54—C55—F1	−176.69 (16)
C4—C3—C7—C8	140.32 (12)	C53—C54—C55—C56	1.7 (3)
C2—C3—C7—C8	−97.48 (14)	C57—C56—C55—C54	−1.0 (3)
C13—C3—C7—C8	23.94 (14)	C57—C56—C55—F1	177.38 (15)
N1—C13—C14—O2	−50.7 (2)	C8—N1—C12—C11	58.8 (2)
C21—C13—C14—O2	−169.40 (15)	C13—N1—C12—C11	−172.14 (16)
C3—C13—C14—O2	64.89 (19)	C15—C20—C19—C24	177.26 (17)
N1—C13—C14—C15	121.82 (14)	C21—C20—C19—C24	0.6 (3)
C21—C13—C14—C15	3.12 (15)	C15—C20—C19—C18	−0.6 (3)
C3—C13—C14—C15	−122.59 (13)	C21—C20—C19—C18	−177.23 (17)
C8—C7—C71—C72	29.5 (2)	N1—C8—C9—C10	54.8 (2)
C3—C7—C71—C72	−93.20 (19)	C7—C8—C9—C10	167.53 (17)
C8—C7—C71—C76	−149.41 (15)	C71—C76—C75—C74	−0.1 (3)
C3—C7—C71—C76	87.91 (17)	C21—C22—C23—C24	2.1 (3)
C4—C5—C51—C52	175.06 (15)	C15—C16—C17—C18	−0.3 (3)
C6—C5—C51—C52	−8.0 (3)	C71—C72—C73—C74	0.5 (3)
C5—C51—C52—C57	−49.1 (2)	C16—C17—C18—C19	−0.4 (3)
C5—C51—C52—C53	136.24 (18)	C24—C19—C18—C17	−176.8 (2)
C15—C20—C21—C22	−174.76 (15)	C20—C19—C18—C17	0.8 (3)
C19—C20—C21—C22	2.2 (2)	C72—C73—C74—C75	−1.1 (3)
C15—C20—C21—C13	2.26 (19)	C72—C73—C74—F2	178.86 (19)
C19—C20—C21—C13	179.18 (15)	C76—C75—C74—C73	1.0 (3)
N1—C13—C21—C22	52.9 (2)	C76—C75—C74—F2	−179.02 (17)
C14—C13—C21—C22	173.26 (17)	N1—C12—C11—C10	−56.4 (2)
C3—C13—C21—C22	−64.5 (2)	C12—C11—C10—C9	55.7 (3)
N1—C13—C21—C20	−123.54 (13)	C8—C9—C10—C11	−54.6 (3)
C14—C13—C21—C20	−3.20 (16)	C22—C23—C24—C19	0.8 (3)
C3—C13—C21—C20	119.04 (14)	C20—C19—C24—C23	−2.1 (3)
C12—N1—C8—C9	−58.33 (18)	C18—C19—C24—C23	175.5 (2)
C13—N1—C8—C9	168.34 (13)		

### Hydrogen-bond geometry (Å, º)

**Table:**

*D*—H···*A*	*D*—H	H···*A*	*D*···*A*	*D*—H···*A*
C11—H11*A*···O1^i^	0.97	2.49	3.352 (2)	148

Symmetry code: (i) *x*−1/2, −*y*+1/2, *z*−1/2.
